# Abnormal Aggregation of Invasive Cancer Cells Induced by Collective Polarization and ECM-Mediated Mechanical Coupling in Coculture Systems

**DOI:** 10.34133/2021/9893131

**Published:** 2021-12-08

**Authors:** Xiaochen Wang, Shaohua Chen, Hanqing Nan, Ruchuan Liu, Yu Ding, Kena Song, Jianwei Shuai, Qihui Fan, Yu Zheng, Fangfu Ye, Yang Jiao, Liyu Liu

**Affiliations:** ^1^Beijing National Laboratory for Condensed Matte Physics, Institute of Physics, Chinese Academy of Sciences, Beijing 100190, China; ^2^School of Physical Sciences, University of Chinese Academy of Sciences, Beijing 100049, China; ^3^Wenzhou Institute, University of Chinese Academy of Sciences, Wenzhou, Zhejiang 325000, China; ^4^Oujiang Laboratory (Zhejiang Lab for Regenerative Medicine, Vision and Brain Health), Wenzhou, Zhejiang 325001, China; ^5^Materials Science and Engineering, Arizona State University, Tempe, Arizona 85287, USA; ^6^Chongqing Key Laboratory of Soft Condensed Matter Physics and Smart Materials, College of Physics, Chongqing University, Chongqing 401331, China; ^7^Department of Physics, Xiamen University, Xiamen 361005, China; ^8^Department of Physics, Arizona State University, Tempe, Arizona 85287, USA

## Abstract

Studies on pattern formation in coculture cell systems can provide insights into many physiological and pathological processes. Here, we investigate how the extracellular matrix (ECM) may influence the patterning in coculture systems. The model coculture system we use is composed of highly motile invasive breast cancer cells, initially mixed with inert nonmetastatic cells on a 2D substrate and covered with a Matrigel layer introduced to mimic ECM. We observe that the invasive cells exhibit persistent centripetal motion and yield abnormal aggregation, rather than random spreading, due to a “collective pulling” effect resulting from ECM-mediated transmission of active contractile forces generated by the polarized migration of the invasive cells along the vertical direction. The mechanism we report may open a new window for the understanding of biological processes that involve multiple types of cells.

## 1. Introduction

Phenotypic and functional heterogeneities arise among cells during development and differentiation, as a consequence of gene expression and environmental changes in a multicellular organism [1]. Spatial separation of genetically distinct clones has also been found in primary tumors [2, 3]. Therefore, cocultures of multiple types of cells have been widely used in *in vitro* studies for tissue formation, cancer, stem cell potency maintenance, etc. [4–7]. In particular, cocultures of subtypes of tumor cells have shown various patterns of cell separation [8, 9]. The dynamics of pattern formation in coculture systems can also provide insights into the cell sorting and patterning of embryogenesis and tumor invasion [10–12].

In studies on coculture systems used to investigate embryogenesis, wound healing, and tissue engineering [13–15], widely implicated is the so-called differential-adhesion hypothesis (DAH), which assumes that a multicellular system can be treated as a Newtonian fluid system. In such a system, when two types of liquids with different surface tensions are mixed together, the final state is given by the requirement that the system has a minimum surface free energy; viz., the two types of liquids would separate, with the type of stronger adhesion staying in the center and that of weaker adhesion staying outside. However, Pawlizak et al. found that DAH was not enough to interpret their experimental observation and suggested that cell mobility should be included as an additional parameter [16].

Moreover, in the DAH, the extracellular matrix (ECM) was not taken into account. Biological processes usually involve coevolution of cells with ECM. Recent experimental and computational studies have revealed that migrating cells in mesenchymal mode in 3D ECM can generate active pulling forces via actin filament contraction within the cells. Such active forces are then transmitted to the ECM network via focal adhesion complexes [17–21] and are able to propagate to an extended range in the ECM, due to the random and network-like nature of the ECM [22–25]. However, the influences of ECM and the long-range forces therein on the patterning of coculture systems remain unexplored.

**Figure 1:**
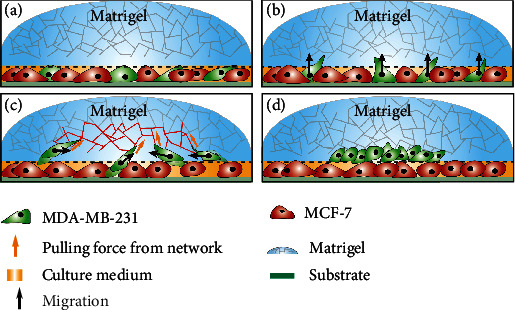
Schematic illustration of the abnormal aggregation of invasive MDA-MB-231 cells induced by collective polarization and ECM-mediated mechanical coupling: (a) MDA-MB-231 cells cocultured with MCF-7 cells covered with a Matrigel layer; (b) MDA-MB-231 cells massively invade into the upper Matrigel region, leading to strong collective polarization; (c) polarized migrating cells generate effective “pulling” forces due to cell contraction via the ECM network; (d) aggregation resulted from the ECM-mediated mechanical coupling among the polarized cells.

In this work, we investigate how ECM may influence the patterning in coculture systems. We design a coculture system composed of highly motile invasive breast cancer cells, initially mixed with inert nonmetastatic cells on a 2D substrate and covered with a Matrigel layer introduced to mimic ECM. This design induced a strongly polarized massive migration of the invasive cells into the upper Matrigel region along the vertical direction, to escape from the overcrowded coculture with the inert cells on the substrate. Along with this massive polarized migration, we observed strong collective and persistent centripetal motion of the invasive cancer cells in the lateral directions, in contrast to random spreading as one might have expected. This mechanism is schematically illustrated in Figure 1. Using additional control experiments, we confirm that the observed aggregation behavior resulted from a “collective pulling” effect in the lateral direction, induced by the Matrigel-mediated transmission of the active contractile forces generated by the polarized migrating cells. Note that this aggregation of motile cancer cells is seemingly contrary to the prediction of DAH. The mechanism we report here, which results from the synergic influences of multiple factors including cell motility and ECM, may shed light on the understanding of many physiological and pathological processes, such as embryogenesis and tumor invasion.

## 2. Results

### 2.1. Rapid Vertical Separation and Abnormal Aggregation

We have built a coculture system containing a mixture of two different types of cells, i.e., highly invasive breast cancer cells (MDA-MB-231) and nonmetastatic cells (MCF-7) on a 2D substrate, which are then covered with a layer of 100% Matrigel (with thickness ∼500 *μ*m). The Matrigel layer provides mechanical support and a microenvironment for the migration of the invasive cancer cells (see Figure 1(a) for illustration and Section 4.2 for details). The invasive MDA-MB-231 cells have low cell-cell adhesion strength (see Table 1 in Sec. III of SI) and high ECM degradation ability [26] and are highly motile both on the 2D substrate and in the 3D ECM. On the other hand, the nonmetastatic MCF-7 cells are very inert, which have very strong cell-cell adhesion and very low motility on the 2D substrate and cannot migrate into the Matrigel layer (see Figure S5 in Sec. III of SI). As shown below, this design can induce strongly polarized migration of the invasive MDA-MB-231 cells into the Matrigel layer.

#### 2.1.1. Strongly Polarized Collective Migration in the Vertical Direction

**Figure 2:**
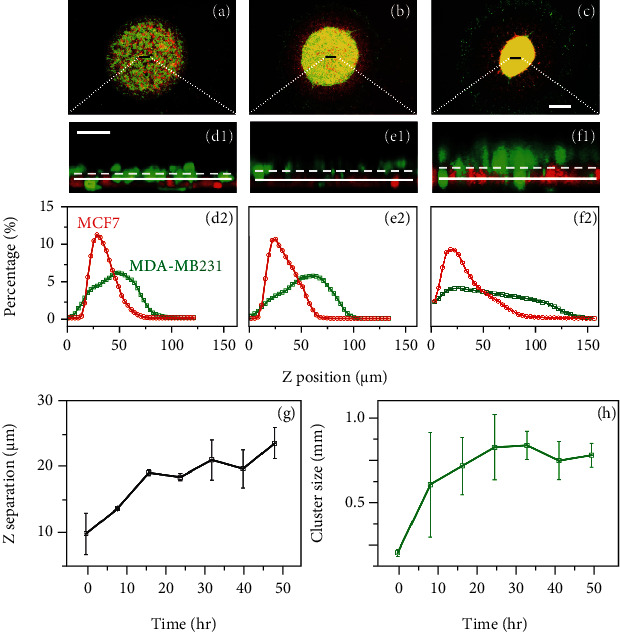
Abnormal aggregation of invasive MDA-MB-231 cells in the lateral directions coupled with collective polarized invasion in the vertical direction. Aggregation dynamics of the MDA-MB-231 cells in this system is also shown at (a) 0 h, (b) 24 h, and (c) 48 h, where the scale bar is 500 *μ*m. (d1)–(f1) show the confocal images (side view, i.e., the *x*-*z* plane; the width of the selected area in the *y*-direction is 50 *μ*m) of systems at (d1) 0 h, (e1) 24 h, and (f1) 48 h, where the scale bar is 50 *μ*m, and the straight lines and the dotted lines represent, respectively, the average positions of MCF7 and MDA-MB231 cells. (d2)–(f2) show the evolution of the distributions of different types of cells along the *z*-direction in the coculture system. (g) shows the average layer separation in the *z*-direction as a function of coculture time; (h) shows the MDA-MB231 colony (cluster) size *L*_c_ of the coculture systems as a function of time, which is strongly correlated with the dynamics of the separation along the *z*-direction. The error bars result from averaging three independent experiments.

We first examined the dynamics along the *z*-direction (perpendicular to the substrate) by using confocal microscopy. Specifically, the distributions of different types of cells along the *z*-direction in the system were computed from time-elapse confocal images and shown in Figures 2(d)–2(f) (see SI Sec. I (1.2) for quantitative methods). It can be clearly seen that a rapid separation between the two cell lines occurred. The majority of the invasive MDA-MB-231 cells migrate into the Matrigel almost simultaneously (SI Sec. II Movies 1 and 2 and Sec. III Figure S3), forming a new layer on top of the MCF-7 layer. We refer to this highly directional massive migration behavior as “collective polarization,” during which each cell can generate strong contractile forces near the front of the cell [12].  Figure 2(g) shows the separation distance *δ* between the average positions of the two types of cells in the coculture system as a function of time, which quantifies the migration dynamics along the *z*-direction. In particular, the invasive MDA-MB-231 cells exhibited strong collective polarization, and the two types of cells quickly separated into two layers. The associated *δ* almost monotonically increased with incubation time.

#### 2.1.2. Abnormal Aggregation in the Lateral Directions

Along with the collective polarized migration, we also observed very strong aggregation dynamics of the invasive cancer cells (Movies 1–3 in Sec. II of SI). As shown in Figures 2(a)–2(c)), starting from a random mixing state with the inert MCF-7 cells, the invasive MDA-MB-231 cells quickly aggregated into a colony (cluster) at the center of the system within the first 24 hours. Afterwards, the MDA-MB-231 cluster continued to shrink towards the center almost isotropically and became highly dense (Figure 2(c)). This is in contrast to random spreading of the invasive cancer cells in the ECM as one might have expected. In addition, the aggregation behavior is also in contrast to the prediction based on the differential adhesion hypothesis (DAH) and thus indicates that the aggregation cannot be cell adhesion dominant.

We employed the two-point correlation function *S*_2_(*r*) [27] to quantify the aggregation patterns associated with the invasive MDA-MB-231 cells and extracted the characteristic length *L*_c_, which is the distance *r* associated with the first local minimum in *S*_2_. The length *L*_c_ is like a correlation length and can be used to characterize the extent of aggregation.  Figure 2(h) shows the evolution of *L*_c_ of the invasive cancer cells as a function of time. It can be clearly seen that *L*_c_ rapidly increased, plateaued at approximately *t* = 24 h, and then slightly decreased. This corresponds to the clustering of the MDA-MB-231 cells in the system (increasing *L*_c_), followed by a further densification of the cluster (decreasing *L*_c_) after 24 hours.

Importantly, it can be clearly seen that the aggregation of the invasive cancer cells was strongly correlated with the collective polarization dynamics shown in Figure 2(h). Specifically, the fastest increase in *L*_c_ of MDA-MB-231 cells (within the first 24 hours), resulting from the “fusion” of individual cells or small clusters into a single large cluster, was accompanied by the massive polarized migration of the MDA-MB-231 cells into the upper Matrigel region. The increases in the length *L*_c_ and the separation *δ* both slowed down after *t* = 24 h. These observations indicate that the collective polarization plays an important role in giving rise to the abnormal aggregation of the invasive MDA-MB-231 cells.

We also mixed fluorescent beads into Matrigel to characterize the gel deformation during the collective cell migration (Movie 2 in Sec. II of SI). The confocal tracking videos clearly showed that MDA-MB231 cells invaded into the gel during the aggregation process and that Matrigel was pulled to the center (in the *x*-*y* plane) while the cells clustered.

### 2.2. Verifying the Role of ECM via Control Experiments

**Figure 3:**
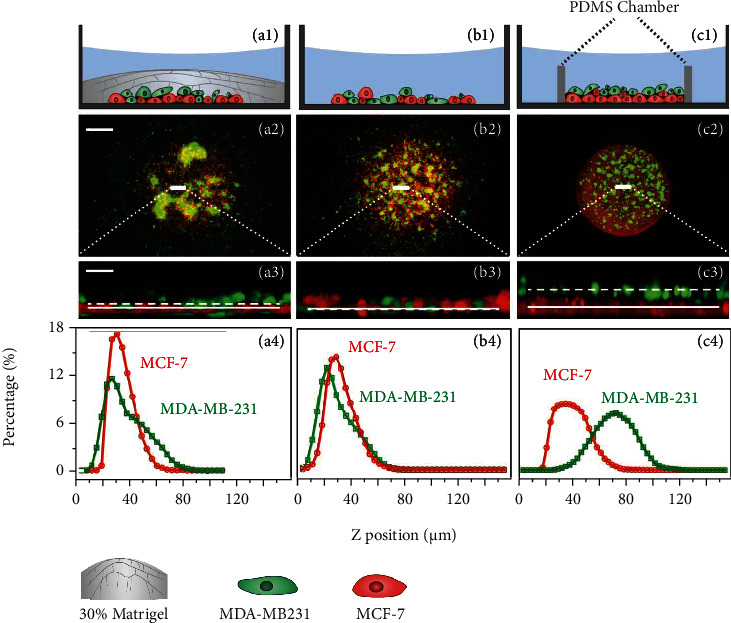
Final aggregation patterns (after 48 h) for different coculture systems as control groups to verify the role of ECM: (a) with 30% Matrigel concentration; (b) without Matrigel layer; and (c) MDA-MB231 cells cultured on top of a layer of MCF7 cells without Matrigel, with the (a1–c1) schematic illustration of different control groups, the top views (a2–c2), and the zoomed-in side views for the small white areas in the top view (a3–c3). The straight lines and the dotted lines in (a3)–(c3) represent, respectively, the average positions of the red cells (MCF7) and green cells (MDA-MB231). (a4)–(c4) show the distributions of different types of cells along the *z*-direction in the coculture system. The scale bars in the first and second row panels are, respectively, 500 *μ*m and 50 *μ*m.

To further elucidate the role of the ECM and collective polarization in the aforementioned observations, we performed several control experiments. We found that reducing Matrigel concentration (e.g., to 30%), which leads to weaker ECM-mediated mechanical coupling, also reduces the separation of two cell lines and thus the aggregation of MDA-MB-231 cells (see Figure 3(a)). In the extreme case that the Matrigel is completely removed (see Figure 3(b) and Figures S2 and S3 in Sec. III of SI), the MDA-MB-231 cells were stuck on the 2D substrate and no collective aggregation was observed. We also performed stratification experiments, in which the two types of cells were initially separated into two layers; viz., the MDA-MB-231 cells were planted on top of the MCF-7 cells; in this case, as shown in Figure 3(c), no strong aggregation was observed either, although the MDA-MB-231 cells remained separated from the MCF-7 cells on the *z*-direction. These experiments proved that the ECM facilitates MDA-MB231 cell's polarized collective migration in the vertical direction and the aggregation in the top layer to form a dense cluster.

### 2.3. Reducing Cell-Cell Adhesion Facilitates Aggregation

**Figure 4:**
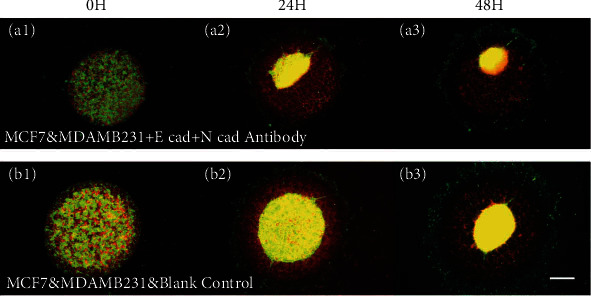
Reducing cell-cell adhesion facilitates MDA-MB231 cells' aggregation. (a1–a3) Pattern evolution of the coculture system with E-cad and N-cad inhibited and (b1–b3) pattern evolution of the control group (with no inhibition), where the scale bar is 500 *μ*m.

As reported in the previous studies about DAH, intercellular adhesion plays a key role in cell segregation [13, 28]. Given that MDA-MB231 cells and MCF7 cells mainly express N-cad and E-cad, respectively (see Table 1 in Sec. III of SI), to act as intercellular adhesion proteins, we added E-cad and N-cad antibodies to the cell culture medium to inhibit the functions of these proteins in the coculture systems. According to the DAH prediction, if E-cad and N-cad were both inhibited, the two types of cells would not separate. However, as shown in Figure 4(a), similar aggregation behavior was observed; moreover, the MDA-MB231 cells aggregated even faster. This enhancement of the aggregation speed by adding E-cad and N-cad antibodies is another strong evidence supporting that the aggregation in our results was indeed induced by the ECM-mediated mechanical coupling rather than by the cell-cell adhesions.

### 2.4. Differences in Cell Mobility Influence Cell Aggregation

**Figure 5:**
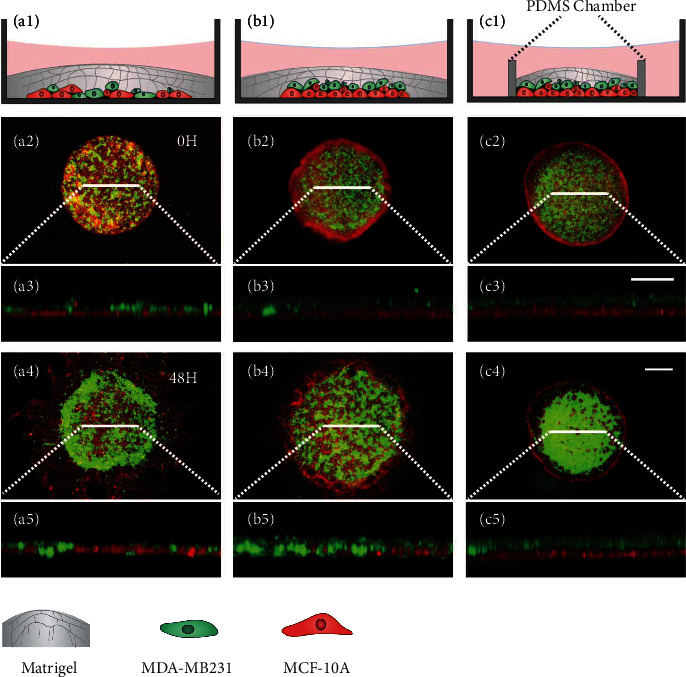
Differences in cell mobility influence coculture cells' segmentation and aggregation. (a1–c1) Schematic illustration of different control groups. High-mobility cell line MCF10A cells (red) were mixed with MDA-MB231 cells (green) under a layer of Matrigel. (a2) and (a3) show, respectively, the top and side views of the coculture system. After 48 h incubation, the MDA-MB231 cells did not aggregate into a dense cluster (a4, a5). When MDA-MB231 cells were seeded on the top of MCF10A cells, after removing PDMS chambers and coating with Matrigel, the two types of cells mixed up in the vertical direction and aggregated into small clusters (b2–b5). (c2–c5) When the coculture system was incubated in a PDMS chamber for 48 h to keep the vertical separation, MDA-MB231 cells tended to form a continuous structure. The scale bars in the vertical and top views are, respectively, 20 *μ*m and 500 *μ*m.

We next investigate how cells' dynamic properties influence their collective segregation and aggregation. We replaced MCF7 cells with the higher-mobility cell line MCF10A (see Figures S2 and S3 in Sec. III of SI), which is benign breast cyst cells. As shown in Figures 5(a1)–5(a5), vertical separation and rapid horizontal aggregation did not occur in the coculture system composed of MCF10A and MDA-MB231 (covered with Matrigel). We then changed the initial condition, starting with MDA-MB231 cells located on top of the MCF10A layer and beneath the Matrigel, and found that the initial stratification was destroyed by the high motility of MCF10A and that the MDA-MB231 cells remained dispersed (Figures 5(b1)–5(b5)); we further changed the boundary condition, by putting an initially stratified system in a PDMS chamber to limit MCF10A cells' migration and found that such confinement did help to maintain the stratification and thus enhanced the aggregation of MDA-MB231 cells (Figures 5(c1)–5(c5)). In the MDA-MB231 and MCF7 cells' coculture system, lower-mobility MCF7 cells provide confinement for MDA-MB231 cells on the 2D plane, which facilitates cells' separation in the *z*-direction. Meanwhile, MCF7 cells also act as a “solid-like substrate” to maintain the stratification and assist the ECM-mediated MDA-MB231 cells' aggregation.

## 3. Discussion

To further investigate the effects of the cell-ECM mechanical coupling on collectively polarized invading cells, we also develop a novel active-particle-on-network model, which explicitly considers the mechanical coupling between distant cells through ECM network-mediated active force propagation [19, 21–23, 29, 30], rather than imposing a simplified effective interaction between nearby cells [31, 32]. The Matrigel is modelled as a nonlinear 3D network with a bond-node representation [21, 29, 33]. The cells in the ECM network, modelled as deformable spheres, can generate active forces by pulling the nodes attached to the cell surface (mimicking focal adhesion sites) via isotropic contraction [22, 33] and sense the total force exerted on the cell (see Figures S6 and S7 for illustration and Sec. IV in SI for details). Thus, the active force generated by a contractile cell can propagate via the ECM network to a distant cell and subsequently influence its migration and vice versa. When multiple cells are present in the 3D ECM network, our model simulates collective migration dynamics regulated by the dynamic force network generated by the actively migrating cells. Visualizations of the evolution of the systems, additional velocity profile analysis, and velocity correlation also verify the validity and accuracy of the proposed model (see Sec. IV Figures S7 and S8). These simulation results support that collective polarization is important in inducing sufficient ECM-mediated mechanical coupling leading to the overall aggregation behavior.

In conclusion, we designed novel experiments which induce strongly polarized massive migration of invasive breast cancer cells into a Matrigel-based ECM and thus enable us to investigate the 3D collective migratory dynamics which are usually masked by complex cellular motion in 3D; our comprehensive experimental and computational investigations indicated that ECM has a significant influence on the patterning of coculture systems. Our results showed that the abnormal aggregation behavior of invasive tumor cells resulted from the strong collective polarization of cell migration and the resulting ECM-mediated mechanical coupling. The influence of metalloprotease (MMP) inhibitors on the aggregation has also been investigated, and the results show that the MMP inhibitors, by reducing the ECM degradation of the cancer cells, significantly slowed down the aggregation rate although they did not prevent the cancer cells from aggregating (see Figure S4 in Sec. III of SI). To the best of our knowledge, this is the first report of direct observation of how ECM influences patterning of coculture systems. Our results indicate the importance of explicitly incorporating the microenvironment into theories for multicellular systems, specifically in explaining collective behaviors of cancer cells during the invasion process.

## 4. Materials and Methods

### 4.1. Cell Cultures

The GFP-tagged-MDA-MB-231 cells were obtained from H. Lee Moffitt Cancer Center, Tampa, FL, USA. The cells were cultured in DMEM medium containing 4.5 g/L glucose and L-glutamine (10-013-CVR, Corning), supplemented with 10% fetal bovine serum (10099-141, Life Technology) and 1% penicillin/streptomycin (30-002-CI, Corning).

The MCF-7 cells (from the American Type Culture Collection (ATCC)) were cultured in MEM containing 1.5 g/L sodium bicarbonate, nonessential amino acid, L-glutamine, and sodium pyruvate (10-009-CVR, Corning), supplemented with 10% fetal bovine serum (10099-141, Life Technology), 10 *μ*g/mL insulin (I-1882, Sigma), and 1% penicillin/streptomycin (30-002-CI, Corning).

The MCF-10A cells (from ATCC) were cultured in DMEM/F12 medium containing L-glutamine and 15 mM HEPES (10-092-CVR, Corning), supplemented with 5% horse serum (16050-122, Gibco), 1% penicillin/streptomycin (30-002-CI, Corning), 20 ng/mL human epidermal growth factor (PHG0311, Gibco), 10 *μ*g/mL insulin (I-1882, Sigma), 100 ng/mL cholera toxin (C-8052, Sigma), and 0.5 *μ*g/mL hydrocortisone (H-0888, Sigma).

All the cell lines were incubated at 37°C with 5% CO_2_. To detach cells from the Petri dish, trypsin (25-053-CI, Corning) and 1x PBS (46-013-CM, Corning) solution (1 : 2 mixture) were applied for 1-2 min. The cells were passaged every 5-6 days for a maximum of 20 passages.

To stain the MCF7 and MCF10A cells, we used CellTracker Orange Red dyes (C34551, Life Technologies), which were first dissolved with 20 *μ*L DMSO (D12345, Life Technology) to 1 mM and later further diluted to the final working concentration of 4 *μ*M in cell suspensions. The cells were then incubated under the growth conditions for 30 min before the solution was replaced by coculture medium.

### 4.2. Coculture Systems

The MDA-MB-231 and MCF-7 cells' coculture medium was DMEM with 10% FBS and 1% penicillin/streptomycin. The MDA-MB-231 and MCF-10A cells' coculture medium was 1 : 1 mixture of the media for monoculture of MDA-MB-231 and MCF-10A cells, so that the epidermal growth factor can be utilized to maintain the proliferation speed of MCF-10A cells. Control groups proved that different coculture media would not influence the main phenomenon reported in the article.

Two types of cells in the coculture systems were mixed at a 1 : 1 ratio with approximately 5∗10^6^ cells/mL and loaded into PDMS chambers (with diameter and height both of 2 mm) attached on Petri dishes, with the culture medium added to the reservoirs. The dishes were then incubated for 18 hours (37°C, 5% CO_2_), so the cells proliferated. After the chambers were completely filled with the cells, the PDMS was torn off and the culture medium was removed. 40 *μ*L of 100% Matrigel was coated on each sample. After Matrigel's crosslinking at 37°C for 30 min, the culture medium was readded.

7 *μ*g/mL E-cadherin (Invitrogen, 13-1700) and 0.5% N-cadherin antibody (Cell Signaling Technology, 13116) were used to regulate cell-cell adhesion.

Fluorescent beads of diameter 2 *μ*m were added in the gel for Matrigel deformation tracking.

### 4.3. Cell Imaging

Fluorescent images were acquired using an inverted microscope Nikon Ti-100 with a digital camera (Canon, EOS700D). The 3D imaging was performed on a confocal microscope (Leica SP8). The fluorescence distribution and multichannel raw images were further processed with MATLAB (MathWorks) and ImageJ (NIH).

### 4.4. Cell and Fluorescent Bead Tracking

3D imaging was performed on a confocal microscope (Leica SP8) working with a homemade live-cell incubating system, which could maintain the culture condition (37°C, 5% CO_2_) for more than 24 hours. The time interval between two scans was 30 min. The process was only recorded for 24 hours by using a HyD photodetector, which is an optimized condition to minimize the optical toxicity.

### 4.5. Statistical Profiles of Cells' Vertical Location Distribution

The confocal 3D reconstructed images were cut along the *x*-*z* plane with a thickness of 50 *μ*m. The *x*-*z* plane screenshots were processed into black (background) and white (cells) pictures, which could be transferred into (0, 1) matrices. Nonzero pixel points were counted along the *x*-axis to obtain a probability distribution of cells at different heights. The mean position of cells was then given by the weighted average of the number of pixels along the *z*-direction (Figure S1).

## Data Availability

All data needed in the paper are present in the paper and in Supplementary Materials. Additional data which are related to this paper may be requested from the authors.

## Supplementary Materials

Supplementary 1 Figure S1: statistical profiles of cells' vertical location distribution. Figure S2: comparison of pattern evolution of different coculture systems. Figure S3: comparison of *z*-separation of different coculture systems. Figure S4: MDA-MB231 and MCF-7 cell coculture with the MMP inhibitor. Figure S5: experiments supporting ECM-mediated mechanical coupling and single-cell dynamics. Figure S6: active-particle model and intercellular force model. Figure S7: simulated cell mixing patterns in different coculture systems. Figure S8: simulated velocity correlation function *C*(*r*) for MDA-MB-231 cells in different microenvironments and with different degrees of collective polarization. Table S1: E-cadherin and N-cadherin expression in three breast cell lines Table S2: summary of the simulation parameters used in our active-particle models.

Supplementary 2 Movie S1: time-lapse movie of MDA-MB231 cells aggregating in the coculture system.

Supplementary 3 Movie S2: time-lapse movie of Matrigel position during MDA-MB231 cell aggregation on a confocal microscope.

Supplementary 4 Movie S3: time-lapse movie of MDA-MB231 cells aggregating in the coculture system on a confocal microscope.
