# Supplementary material for: Abnormal Aggregation of Invasive Cancer Cells Induced by Collective Polarization and ECM-Mediated Mechanical Coupling in Coculture Systems
Source: Research (Wash D C). 2021 Dec 8;2021:9893131. doi: 10.34133/2021/9893131 (PMC8678614; doi:10.34133/2021/9893131)
Supplement: Supplementary 1 — Figure S1: statistical profiles of cells' vertical location distribution. Figure S2: comparison of pattern evolution of different coculture systems. Figure S3: comparison of z-separation of different coculture systems. Figure S4: MDA-MB231 and MCF-7 cell coculture with the MMP inhibitor. Figure S5: experiments supporting ECM-mediated mechanical coupling and single-cell dynamics. Figure S6: active-particle model and intercellular force model. Figure S7: simulated cell mixing patterns in different coculture systems. Figure S8: simulated velocity correlation function C(r) for MDA-MB-231 cells in different microenvironments and with different degrees of collective polarization. Table S1: E-cadherin and N-cadherin expression in three breast cell lines Table S2: summary of the simulation parameters used in our active-particle models. [file 9893131.f1.docx]

Supplementary Materials for

Abnormal Aggregation of Invasive Cancer Cells Induced by Collective Polarization and ECM-mediated Mechanical Coupling in Co-culture Systems

Xiaochen. Wang, Shaohua. Chen, Hanqing. Nan, Ruchuan. Liu, Yu. Ding,

Kena. Song, Jianwei. Shuai, Qihui. Fan, Yu. Zheng,

Fangfu. Ye,^*^ Yang. Jiao,^*^ Liyu. Liu,^*^

*Corresponding author. Email: lyliu@cqu.edu.cn(L.L.); yang.jiao.2@asu.edu(Y.J.); fye@iphy.ac.cn(F.Y.)

**This PDF file includes:**

Supplementary Text

Figs. S1 to S8

Tables S1 to S2

Movies S1 to S3

References

**Other Supplementary Materials for this manuscript include the following:**

Movies S1 to S3

Supplementary Text

**Section I Experimental Methods**

**1.1 Cell Tracking**

(1) Cell force field tracking

We prepared a 3D device for force field tracking (see Liu et al (*35*) for details of the device design). The cells were mixed with 4.7mg/mL Collagen rather than Matrigel, given that the stiffness of the latter is too high to be deformed by individual cells. Fluorescent beads of diameter 2μm were added in the gel, and their confocal images were collected every 12 hours.

(2) Cell tracking in low cell-density systems for MSD and correlation analysis

Cells were diluted into 1×10^4^ cells/mL and cultured on the Petri dishes. After the cells attached on the dish (incubated for 18 hours at 37℃, 5% CO_2_), the samples were coated by 100% Matrigel. In the blank control groups, new culture medium was added directly. A Nikon Ti-E inverted microscope with a phase contrast 10X objective (Nikon) was used to record the cells’ migration through a digital camera (Hamamatsu, C11440). An incubation system for microscope (Tokai Hit) was used to maintain culture conditions (37℃, 5% CO_2_) for cells on the microscope. Images were collected every 2 mins for more than 8 hours (with the corresponding results given in Fig. S5).

(3) Cell tracking in high cell-density systems for density distribution and velocity field analysis

3-D imaging was performed on a confocal microscope (Leica SP8) working with a homemade live-cell incubating system, which could maintain the culture condition (37 ℃, 5% CO_2_) for more than 24 hours. The time interval between two scans was 30 mins. The process was only recorded for 24 hours by using HyD photodetector, which is an optimized condition to minimize the optical toxicity. To obtain a bigger view, we used 10X air objective lens and marked three positions along the central axis on the x-y plane to merge images. The corresponding cell density distribution and velocity field can then be calculated accordingly, with the results given in Fig. S7.

**1.2 Statistical Profiles of Cells’ Vertical Location Distribution**

The confocal 3D reconstructed images are cut along the x-z plane with a thickness of 50μm. The x-z plane screenshots are processed into black (background) and white (cells) pictures, which could be transferred into (0, 1) matrices. Non-zero pixel points are counted along the x-axis to obtain a probability distribution of cells at different heights. The mean position of cells is then given by the weighted average of the number of pixels along the z
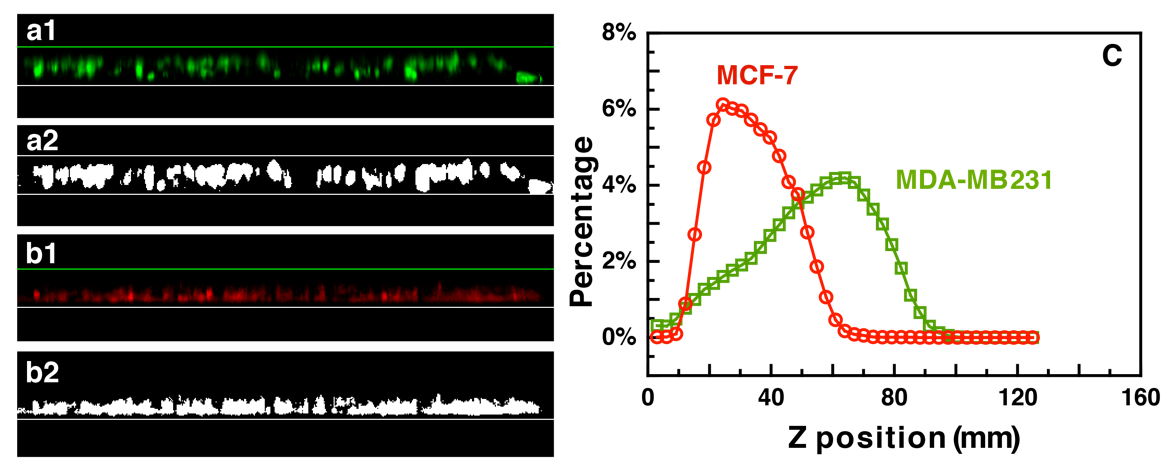
direction.

**Figure S1: Statistical Profiles of Cells’ Vertical Location Distribution.** The reconstructed confocal images (on the x-z plane) of MDA-MB231 (a1) and MCF7 (b1) are processed into black-white pictures (a2, b2); and (c) gives the vertical location distribution of two types of cells.

**1.3 Calculation of Mean Squared Displacements and Velocity Correlations**

iTrack4U software was used to track the time-dependent positions of the centroids of individual cells in 2D low cell density systems (*36*). Given the positions [*x*(*t*), *y*(*t*)] of cells, the mean-squared displacements (MSDs) can then be calculated(*37*) ,


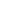
 $\mathrm{MSD}\left( n\cdot\Delta t \right)=\frac{1}{N-n+1}\sum_{i=0}^{N-n} \left[ {(x_{\left( i+n \right)\Delta t}-x_{i\Delta t})}^{2}+{(y_{\left( i+n \right)\Delta t}-y_{i\Delta t})}^{2} \right]$ (S1)

where Δ*t* is the minimal time interval (between two images), *n* represents the number of time intervals, *N* is the total number of time intervals per trajectory.

The velocity correlation function is given by the average of the inner product of the unit vectors representing the instant velocity directions of cells *i* and *j*,

$C\left( r \right)=\left\langle\frac{v_{i}\cdot v_{j}}{\left| v_{i} \right|\cdot\left| v_{j} \right|} \right\rangle$ (S2)

where < ∙ > denotes the average and *r* is the distance between the cells. We performed three independent experiments. In each experiment, we selected 40 pairs of cells for each given distance *r* to compute the averaged value of their correlations, and this value is then further averaged over the 3 independent experiments. The error bars shown in Fig. S5(b) in the main text are associated with the second averaging.

**Section II Supplementary Movies**

**Supplementary Movie1: Time-lapse movie of MDA-MB231 cells aggregating in the co-culture system.**

Time-lapse movie of the MDA-MB231 cells’ rapid aggregation when co-cultured with MCF-7 cells shows the whole process of the cells’ behaviors in the quasi-three-dimensional co-culture system. The images were taken every 10mins on the inverted microscope (Nikon) with the live stage micro-incubator. It shows that the aggregation process could be divided into two steps: i) small groups aggregating into larger ones, ii) cluster shrinking. One second in the video represents 75mins.

**Supplementary Movie2: Time-lapse movie of Matrigel positon during MDA-MB231 cell aggregation on confocal microscope.**

Because Matrigel cannot be observed directly on a confocal microscope, fluorescent beads (blue) of 1μm diameter were mixed into Matrigel to characterize the position of gel. MDA-MB231 cells are green and MCF7 cells are red. The images were taken every 30mins with the live stage micro-incubator. One second in the video represents 120mins, and the tracking time is 24 hours.

**Supplementary Movie3: Time-lapse movie of MDA-MB231 cells aggregating in the co-culture system on confocal microscope.**

Time-lapse movie of the MDA-MB231 cells’ rapid aggregation was recorded by the confocal microscope. And then each cell was tracked and the trajectories were further quantitatively analyzed. The images were taken every 30mins with the live stage micro-incubator. One second in the video represents 120mins.

**Section III Supplementary Experiments**

**Supplementary Table1: E-cadherin and N-cadherin expression in three breast cell lines**

| **Cell lines** | **E-cadherin** | **N-cadherin** |
| --- | --- | --- |
| MCF7 | 1 | 1 |
| MDA-MB231-GFP | 0.0188 | 3.497 |
| MCF10A | 5.089 | 0.4851 |

The E-cadherin and N-cadherin expressions are studied by qPCR. The expressions of cadherin in MCF7, MDA-MB231-GFP, and MCF10A cells are normalized to GADPH, with the value for MCF7 cells set to be 1.

**Supplementary Figure S2: Comparison of pattern evolution of different co-culture systems**


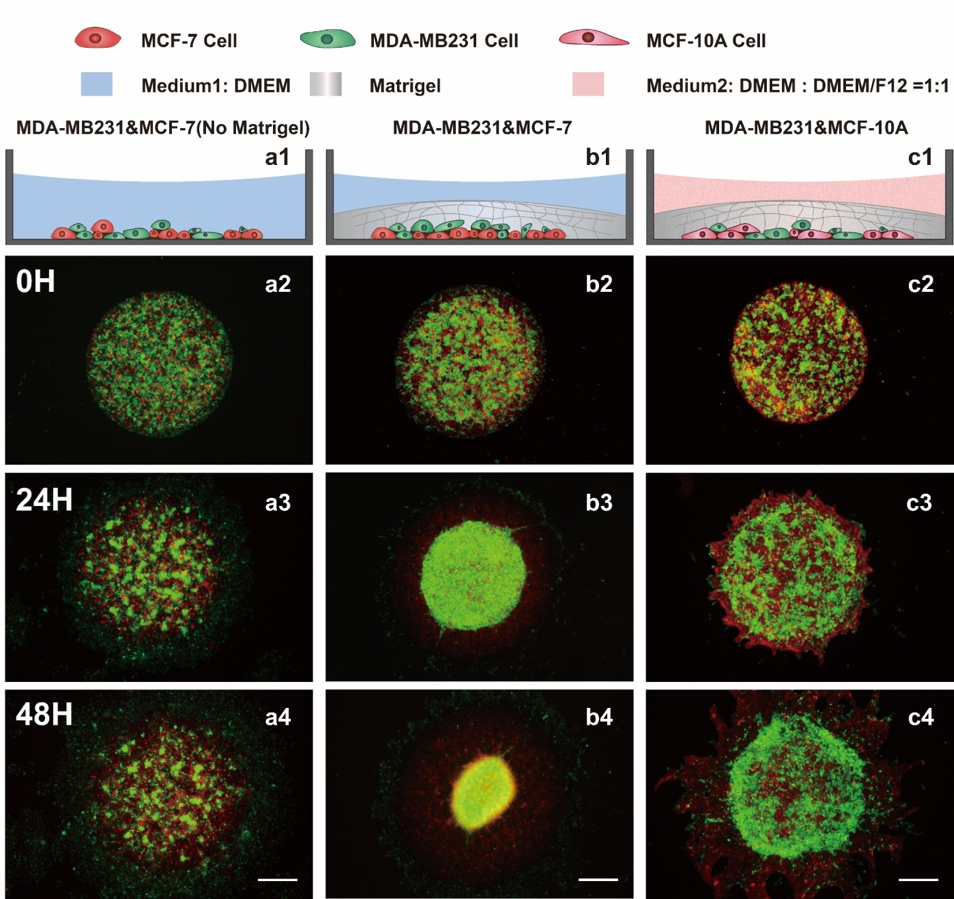


**Figure S2: Comparison of pattern evolution of different co-culture systems**: (a) MDA-MB-231 cells (green) co-cultured with MCF-7 cells (red) on Petri dish, without Matrigel coating; (b) similar to (a) with Matrigel coating; (c) similar to (b) while MCF-7 cells are replaced by MCF-10A cells (red). Without Matrigel coating, the MDA-MB231 cells aggregate into small spots spreading all over the Petri dish, surrounded by the MCF-7 cells. Once coated by Matrigel, the MDA-MB-231 cells rapidly aggregate into one large spot, gradually shrinking in size and becoming denser. However, if the MDA-MB-231 cells are co-cultured with MCF-10A cells and also coated by Matrigel, similar aggregation phenomenon is absent. Here the 18th hour after seeding the cells is defined as 0H. The white scale bar in the figures is 500μm.

**
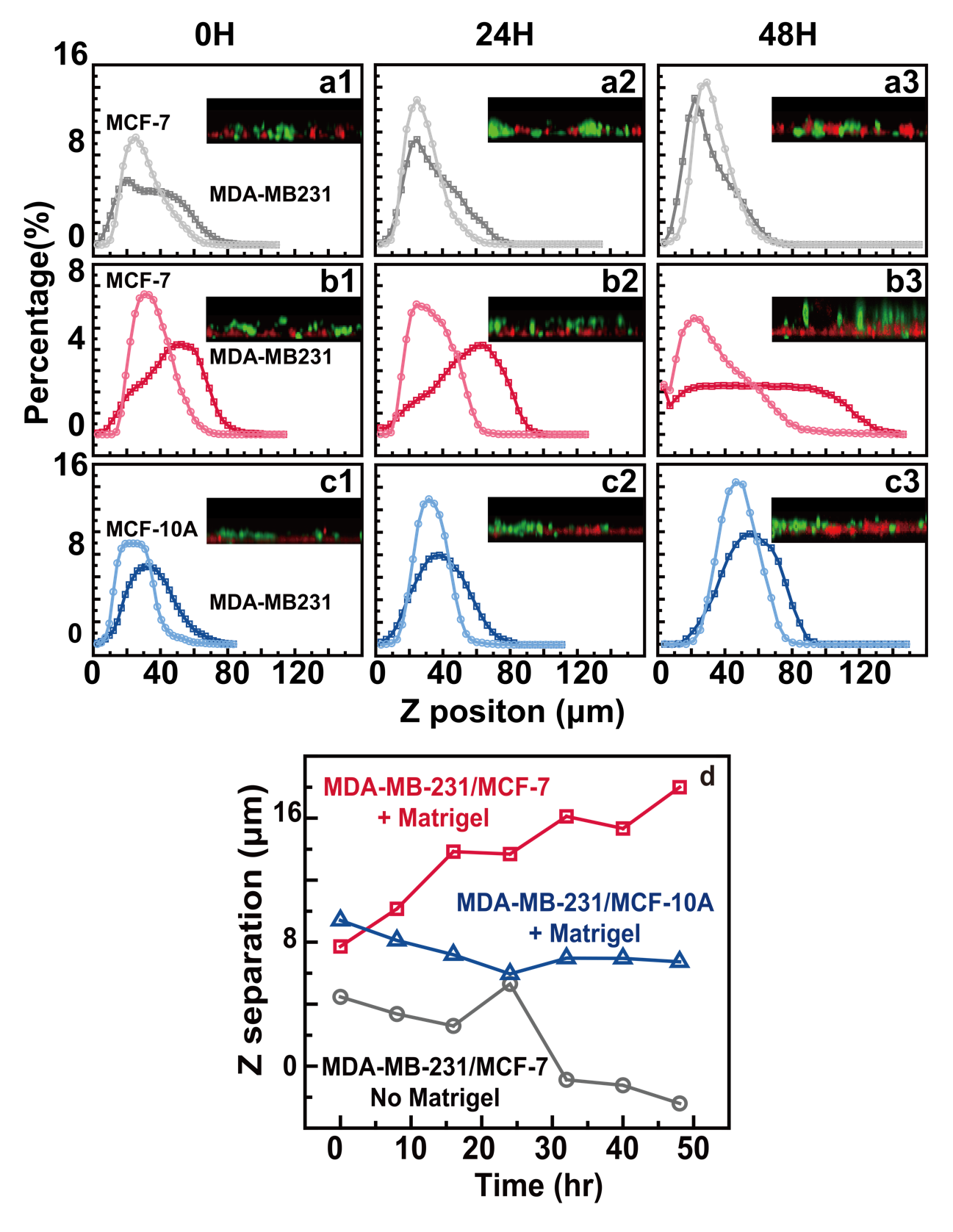
Supplementary Figure S3: Comparison of z-separation of different co-culture systems**

**Figure S3: Comparison of z-separation of different co-culture systems.** (a-c) Evolution of the distribution of different types of breast cell lines along the z-direction in the co-culture systems: (a1-a3) MDA-MB-231+MCF7 without Matrigel, (b1-b3) MDA-MB-231+MCF7+Matrigel, (c1-c3) MDA-MB-231+MCF10A+Matrigel, where the inserted frames are the side-view confocal images. (d) Separation between two types of co-cultured cells, as quantified by the difference between their mean positions along the z-direction.

**Supplementary Figure S4: MDA-MB231 and MCF-7 cells co-culture with MMPs inhibitor**

**
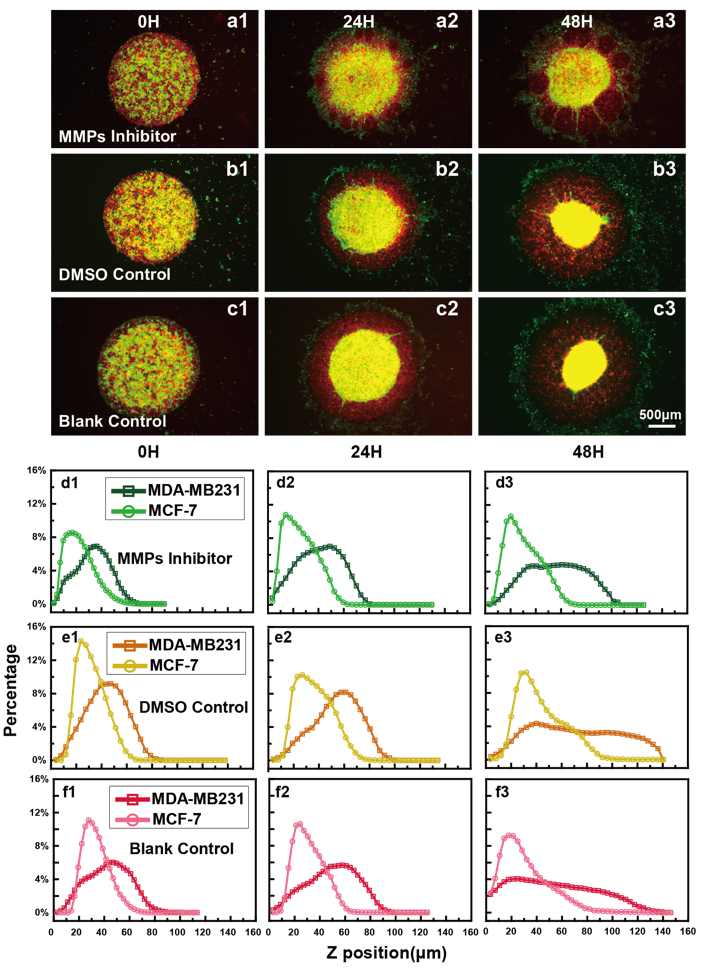
**

**Figure S4 MDA-MB231 and MCF-7 cells co-culture with MMPs inhibitor**: (a-c) Aggregation processes of the MMPs inhibitor group [MDA-MB231+MCF7+Matrigel co-culture system treated by 40μM broad-spectrum metalloproteases (MMPs) inhibitor (Batimastat, Millipore 196440)], the DMSO control group (MDA-MB231+MCF7+Matrigel treated by DMSO), and the blank control group (MDA-MB-231+MCF7+Matrigel, no inhibitor or DMSO), respectively; (e-g) corresponding evolutions of the cells distributions along the z-direction. The MMPs inhibitor reduced the ECM degradation by MDA-MB-231 cells and thus retarded the aggregation process. The DMSO control experiments were performed as well, given that DMSO is the solution for the MMPs inhibitor; the corresponding results show that DMSO has negligible effects on the aggregation.

**Supplementary Figure S5: Experiments supporting ECM-mediated mechanical coupling and single cell dynamics of different types of cells**

We now proceed to further understand the mechanisms governing the strong correlation between collective polarization and the abnormal aggregation. We first used fluorescent beads to track the gel deformation induced by active contraction of migrating individual MDA-MB-231 cells. As shown in Fig. S5(a), the vertical migration of a cell can lead to significant contractile deformation in the surrounding gel in the lateral directions, suggesting that during strongly polarized migration in the vertical direction the cells are effectively “pulling” one another in the lateral directions via the ECM network. We argue that this ECM-mediated mechanical coupling in turn results in the observed lateral aggregation.

We then investigate how ECM influences the cells’ velocity correlation in systems of low cellular density (with a cell covering fraction of ∼1% in the image). We used high-resolution time-elapse microscopy to track each individual cell, and then mapped their trajectories and computed the associated migration velocities v (see Fig. S5 in SI Sec. III). The velocity correlation function is given by C(r) =< (vi · vj )/(|vi||vj |)>, where < · > denotes the ensemble average of the inner product of the instant velocities of a pair of cells (indexed by i and j) separated by distance r. Fig. 6(b) shows C(r) of MDA-MB-231 on the 2D substrate with and without Matrigel in the low-density systems and the associated exponential fittings, i.e., C(r)∼exp(−r/a), where a represents the dynamic correlation length [see Sec. I (1.2) in SI for details]. It can be seen that the MDA-MB-231 cells with Matrigel show a stronger dynamic correlation, presumably due to the ECM-mediated mechanical coupling, as indicated by the slower decay of C(r) or equivalently the larger correlation length: a≈20µm for those with Matrigel while a≈12µm for those without.


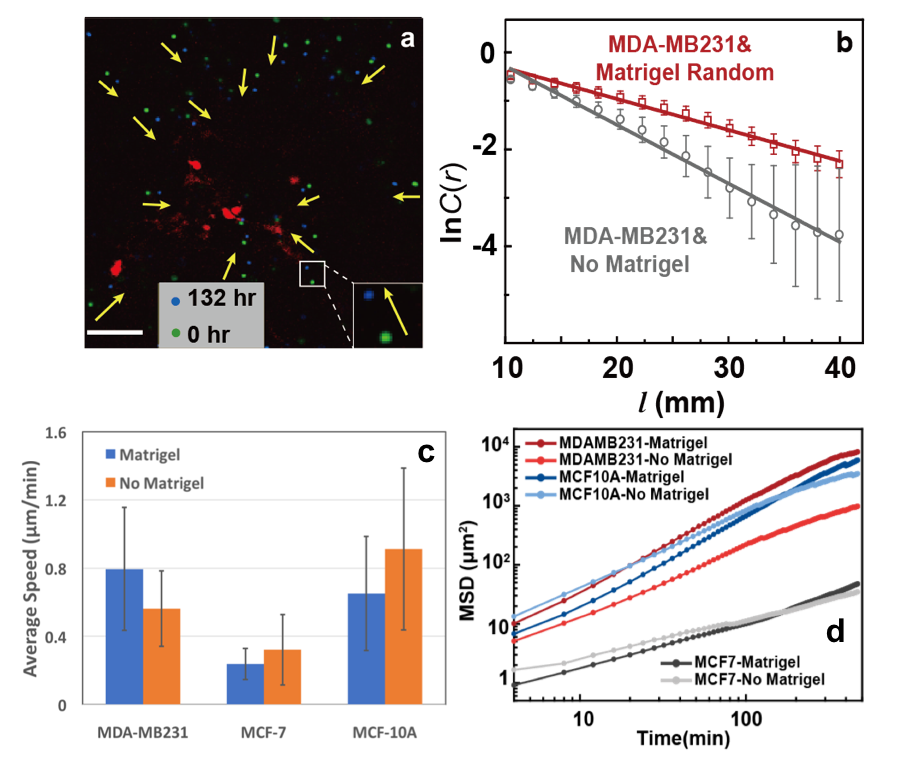


**Figure S5. Experiments supporting ECM-mediated mechanical coupling and single cell dynamics of different types of cells:** (a) Deformation field in the lateral directions in 3D Matrigel caused by active contraction of migrating MDA-MB-231 cells in the vertical direction, where the yellow arrows represent the measured displacements of imbedded fluorescent beads in 132 hours. (b) Experimentally measured velocity correlation function C(r) for MDA-MB-231 cells in different micro-environments at low cell densities, where the grey line with a slope of -0.080 is for the system with no Matrigel and the red line with a slope of –0.048 is for the system covered with Matrigel, in which the cells migrated almost randomly with no collective polarization. The error bars result from averaging over 3 independent experiments. The inverses of the slopes then give the correlation lengths, which are ~12μm and ~20μm respectively. Experimentally measured single cell dynamics of different types of cells in different micro-environment: (c) average velocity; (d) mean square displace (MSD).

**Section IV Computational Methods and Results**

**4.1 Active-particle model incorporating cell-ECM mechanical coupling**

In our model, the cell motion follows the over-damped Langevin equation

$\frac{d\mathbf{r}_{i}(t)}{dt}=\mu_{i}\left[\sum_{j} \mathbf{F}_{ji}(t)+\mathbf{F}_{ECM}(t)+f_{i}\mathbf{e}_{i}(t)\right]+\eta_{i}\mathbf{u}_{rand}$ (S3)

where r_i_ is the position vector of cell i, fi is associated with the persistent velocity (i.e., cell motility) depending on the micro-environment (e.g. 2D substrate vs. 3D Matrigel) and the velocity direction is specified by the unit vector ei, which is subject to rotational diffusion, u_rand_ is a random unit vector, F_ji_ is the inter-cellular force between cells i and j upon contact, FECM is the total force that ECM exerts on the cell and is only non-zero for cells in the ECM region, µ_i_ and η_i_ characterize, respectively, the cell’s responses to external forces and random drift. The cellular motilities for different types of cells in different micro-environments are calibrated based on experimentally obtained individual cell dynamics. The inter-cellular contact force F_ji_ is repulsive when the two cells are very close to one another (due to cytoplasm exclusion volume), which decays to zero as the cell-center distance increases and then becomes adhesive for a finite range [see 4.2 for details] (*32*).

The Matrigel is modelled as a nonlinear 3D network with a bond-node representation (*23, 24, 30*). The cells in the ECM network, modelled as deformable spheres, can generate active forces by pulling the nodes attached to the cell surface (mimicking focal adhesion sites) via isotropic contraction (*19*), and sense the total force F_ECM_ exerted on the cell [see 4.4 for details]. Thus, the active force generated by a contractile cell can propagate via the ECM network to a distant cell and subsequently influence its migration and vice versa. When multiple cells are present in the 3D ECM network, our model simulates collective migration dynamics regulated by the dynamic force network generated by the actively migrating cells.

**4.2 Intercellular force model and dynamic parameters**

We have employed an active-particle model to investigate the collective aggregation behavior in the co-culture systems. The key model parameters and their associated biophysical mechanisms have been discussed in the main text. The inter-cellular force between neighboring cells i and j in Eq. (S3) is given by

$F_{ij}=\left\{ \begin{aligned} k_{ij}\left( R_{i}^{0}+R_{j}^{0}-r_{ij} \right), r_{ij}<\left[ R_{i}^{0}+R_{j}^{0} \right] \\ \beta_{ij}\left( R_{i}^{0}+R_{j}^{0}-r_{ij} \right), \left[ R_{i}^{0}+R_{j}^{0} \right]<r_{ij}<\left[ R_{i}^{1}+R_{j}^{1} \right] \\ \beta_{ij}\left[ \left( R_{i}^{0}+R_{j}^{0} \right)-2\left( R_{i}^{1}+R_{j}^{1} \right)+r_{ij} \right], \left[ R_{i}^{1}+R_{j}^{1} \right]<r_{ij}<\left[ 2\left( R_{i}^{1}+R_{j}^{1} \right)-\left( R_{i}^{0}+R_{j}^{0} \right) \right] \\ 0, r_{ij}>\left[ 2\left( R_{i}^{1}+R_{j}^{1} \right)-\left( R_{i}^{0}+R_{j}^{0} \right) \right] \end{aligned} \right.$ （S4）

where r_ij_ is the separation between cell i and j, and R_0_ and R_1_ are, respectively, the exclusion sphere radius and the effective range of adhesion. We note this form of inter-cellular interaction includes a strong repulsion when the cells are very close to one another. The repulsive force decreases as the separation distance increases, and eventually becomes an adhesive force, which first increases and reaches a maximal magnitude and then decreases as the separation distance further increases. Cells that are well separated do not exert direct mechanical forces on one another.

The simulation domain is a cuboid with dimensions 2mm by 2mm by 50µm along the x, y and z axis respectively, with hard-wall boundary conditions. Initially, 5000 MDA-MB-231 cells and 5000 MCF-7 (or MCF-10A) cells are randomly distributed in a circular region with diameter 1 mm in the *x-y* plane. For all types of cells in the mixture, we use *R*^0^ = 10µm and *R*^1^ = 1.5*R*^0^ = 15µm in Eq. (S1). We use *R*^0^ as the length unit and Δ*t* = 1 (i.e., a single time step, which is roughly 10mins) as time unit in our simulations, both of which can be easily re-scaled for quantitative comparison of experimental and computational results. Without loss of generality, we set the dimensionless parameters *µ*=*η*=1 for all cell types [c.f. Eq. (S3)], which implies that different cells have similar response to the same forces and fluctuations. The cell motility *f*=0.25R^0^, 0.15R^0^ and 0.01R^0^ per Δ*t*, respectively, for MDA-MB-231, MCF-10A and MCF-7 cells. The repulsion strength *κ* = 20/Δ*t* for all cell types, implying strong cytoplasm exclusion volume effect. The adhesion strength *β*=1.05/Δ*t*, 3.8/Δ*t,* and 3.8/Δ*t* respectively for MDA-MB-231, MCF-10A and MCF-7 cells for same type interactions, and 1.85/Δ*t* for MDA-MB-231/MCF-7 and 3.05/Δ*t* for MDA-MB-231/MCF-10A. The particular choices of the model parameter values are based on our experimental observations and calibrated so that the simulation results quantitatively agree with the corresponding experimental results. We note that a range of values for the key dynamics parameters *f*, *κ* and *β* are investigated. The reported values are those that repeatedly lead to stable collective behaviors over reasonable time scales comparable to the experimental time scale (e.g., ~ 48 hours). The modeling parameters are also summarized in the table below.

**Table 2:** **Summary of the simulation parameters used in our active particle models.**

| **Mechanisms** | **Model Parameters** | **MDA-MB-231** | **MCF-7** | **MCF-10A** |
| --- | --- | --- | --- | --- |
| **Overall mobility** | *f***, µ, η** | **(0.25R^0^/Δ*t,* 1, 1)** | **(0.01R^0^/Δ*t,* 1, 1)** | **(0.15R^0^/Δ*t,* 1, 1)** |
| **Repulsion strength** | ***κ*** | **20/Δ*t*** | **20/Δ*t*** | **20/Δ*t*** |
| **Adhesion strength** | ***β*** | **1.05/Δ*t*** | **3.8/Δ*t*** | **3.8/Δ*t*** |


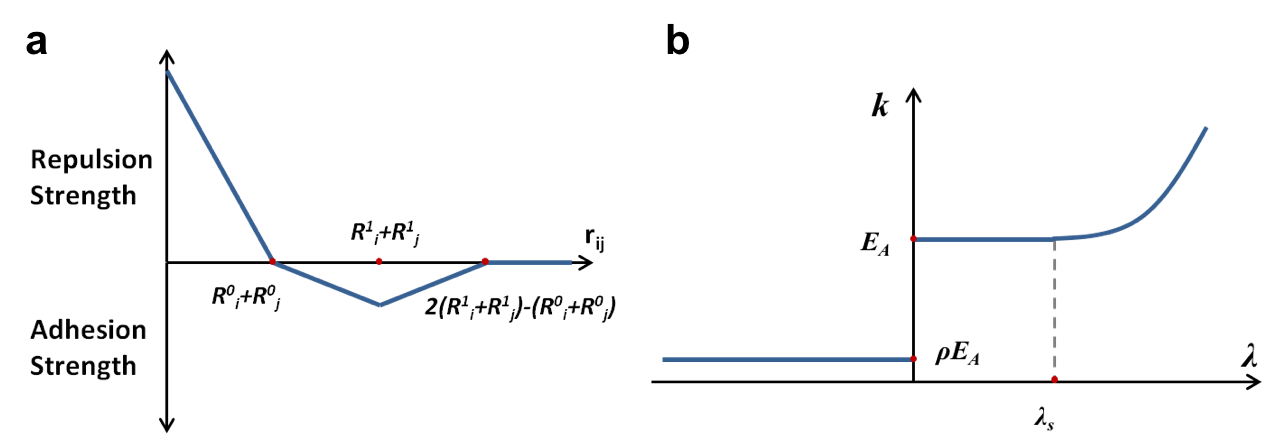


**Figure S6: Active-particle model (a) and Intercellular force model (b)**

**4.3 Simulated aggregation dynamics in the co-culture systems**

In the main paper, we have briefly reported the aggregation dynamics of various co-culture systems obtained from our simulations, which are in excellent agreement with the corresponding experimental results. Here, we provide additional results of the simulated aggregation dynamics. In the simulation of co-culture system without the Matrigel top [Fig. S7(a1-1a4)], a large number of intercellular contacts and strong adhesions between cells are found. Due to the low mobility, the MCF-7 cells effectively serve as “obstacles” for the migration of MDA-MB-231 cells, leading to very limited aggregation of both types of cells and the slow growth of the colony size in the system.

Fig. S7(b1-b4) show that, by adding the Matrigel top onto the MDA-MB-231/MCF-7 co-culture system, the strong ECM degradation ability of the MDA-MB-231cells leads to a rapid separation of the two types of cells along the *z* axis, as the MDA-MB-231cells originally jammed in the *x-y* plane are quickly “squeezed” into the Matrigel. This helps to eliminate the “obstacles” as in the 2D co-culture system, allowing the large-scale aggregation of MDA-MB-231cells towards the system center. We emphasize that such aggregation is resulted from the ECM induced collectiveness.

However, if the “inert” MCF-7 cells in the system is replace by the highly mobile and more adhesive MCF-10A cells, additional degree of freedom for migration in the *x-y* plane due to the quickly moving away of MCF-10A cells in the outer rim significantly reduces the motion of MDA-MB-231 cells in *z*-axis as well as the *z*-separation of two cell lines. This leads to a lower degree of colonizing and aggregation of MDA-MB-231 cells, as shown in Fig. S7(c1-c4).

**4.4 Micromechanics of ECM network**

The ECM network is modeled using a bond-node representation. For the 100% Matrigel used in the experiments, the average bond length in our model is 2.6µm, each node on average connects to 3.8 bonds and the average node number density is 1.3µm^-3^. Following Ref. (*20*), we employed a nonlinear micro-mechanical model for the bond segments, which buckle upon compression and exhibit strain-hardening upon stretching. Specifically, the elongation stiffness *k* of a bond is given by:

$k=\left\{ \begin{aligned} \rho E_{A}, \lambda<0 \\ E_{A}, 0<\lambda<\lambda_{s} \\ E_{A}\exp\left[ \left( \lambda-\lambda_{s} \right)/\lambda_{0} \right], \lambda>\lambda_{s} \end{aligned} \right.$ (S4)

where E_A_ = 8*10^-7^N, λ is the elongation strain, λs = 0.02 and λ_0_ = 0.05 are strain-hardening parameters; *ρ*=0.1 describes the effects of buckling. In addition, the bond also possesses a bending modulus E_I_ = 5*10^-10^Nµm^2^. The average Young’s modulus of the model ECM network is ~100Pa, consistent with the experimentally measured modulus reported in Ref.(*24*).

Following Ref.(*20*), each cell in ECM network is modeled as a sphere with radius *R^0^*. The nodes whose distances to the cell center are smaller than *R^0^* are considered attached to the cell via focal adhesion [see Fig. S7(d)]. At each time step, the cells (i.e., spheres) in the ECM network perform a contraction characterized by the volume strain *ϵ*=-0.05, and pull the bonds connected to the cells. This leads to an average pulling force of ~100nN, consistent with the experimentally measured force generated by contractile migrating MDA-MB-231 cells in Matrigel reported in Ref.(*24*). The total force that ECM exerts on the cell is computed as the vector sum of the forces on the fibers connected to the cell. ECM degradation by MDA-MB-231 cells is then modeled by removal of randomly selected fibers connecting to the cells with a probability *p*_d_ = 0.05 at each time step. The active pulling forces generated by a single cell propagate through the network fibers, which form a dynamically evolving force network depending on the configuration of the cells [see Fig. S7(e)]. Such force network regulates the migration of the cells according to Eq. (S3) in 4.1.

In Fig. S7, we briefly illustrate the force field generated by the active cells in the ECM. Fig. S7(d) shows a schematic illustration of the mechanical coupling between a contractile cell and the fibrous ECM, in which the nodes connected to the cell are pulled towards the center of the cell as it contracts. The resulting force can propagate to a distant cell, offering a possible mechanism for long-range mechanical signaling. Fig S7(e) shows the “force chains” formed between two contracting cells. Fig. S7(f1-f3) illustrates the evolution of force field in the cell-ECM system due to the contraction of the cells, which eventually leads to the aggregation of the MDA-MB-231 cells in the ECM. The force field shown is a coarse-grained representation of the discrete force network, in which dark color indicates pulling forces between cells.


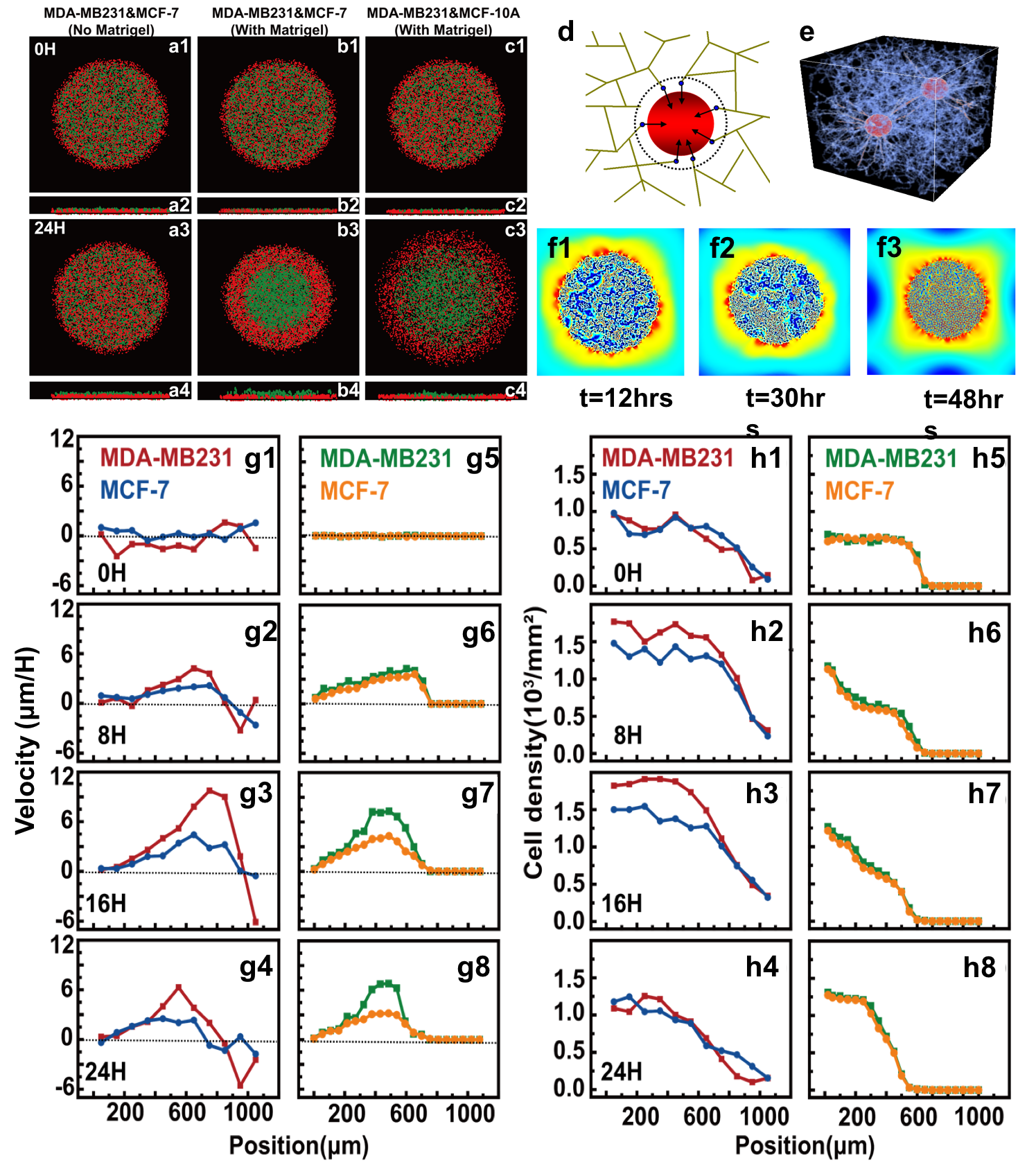


**Figure S7: Simulated cell mixing patterns in different co-culture systems**: (a) MDA-MB231 co-cultured with MCF-7, with no Matrigel; (b) MDA-MB231 co-cultured with MCF-7, with Matrigel covered on top; (c) MDA-MB231 co-cultured with MCF-10A, with Matrigel covered on top. (**a1-c1**) respectively show the initial configurations of the co-culture systems, while (**a2-c2**) show the corresponding cross sections in the *x-z* plane; (**a3-c3**) respectively show the final configurations of the co-culture systems (at *t*=24 hours), while (**a4-c4**) show the corresponding cross sections in the *x-z* plane. **Illustration of the force field generated by active contraction of cells in ECM.** (d) Schematic illustration of the mechanical coupling between a contractile cell and the fibrous ECM. (e) The “force chains” (highlighted with red color) formed between two contracting cells. **(f1-f3) Evolution of force field in the cell-ECM system due to the contraction of the cells, which eventually leads to the aggregation of the MDA-MB-231 cells in the ECM.** The color indicates the relative magnitude of the tensile/pulling forces in the system, and red color indicates larger forces while blue color indicates smaller forces. It can be seen that as the cell cluster becomes denser, it generates almost uniform pulling force at the cluster-ECM interface, indicating the cluster is contracting. **Cell velocity field and cell distribution of MDA-MB-231/MCF-7/Matrigel system from cell tracking on the confocal microscope and simulation results.** (g1-g4) Experimental velocity projection in the Y direction of cells is plotted as a function of cell position. The horizontal dotted lines separate cells migration directions: the part above the line is for cells moving toward the center (*v* > 0); below the line for cells leaving the center (*v* < 0). (g5-g8) Averaged radial velocity profiles during the aggregation obtained from the active particle simulations. (h1-h4) Experimentally measured cell density as a function of distance from the system center.; (h5-h8) Angularly averaged cell number density along the radial direction obtained from the active particle simulations. The coherence of the profiles for the two cell lines indicates correlated motions.

**4.5 Comparison between experimental and simulation results on cell dynamics**

We now present the detailed analysis of the dynamics of the MDA-MB-231 and MCF-7 co-culture system coated with Matrigel. Time-lapse confocal microscopy and single-cell tracking software were applied to investigate cell velocity field and cell distribution. We choose a 2.5mm×0.9mm area along the radial (see Supplementary Movie3). The velocity field is the distribution of velocity projection in the Y direction. Within the first 8 hours, MDA-MB-231 cells moved randomly and gathered into small groups. After that, small groups moved to form larger clusters, and cells had a distinct tendency aggregating to the center. Approximately speaking, the cells’ velocity is proportional to their distance to the center, and the cells have a higher density in the central regions. The results are shown in Fig. S7 (g1-g4) & (h1-h4).

For purpose of comparison, we also computed the angularly averaged cell number density along the radial direction and the averaged radial component of the cell migration velocity for both types of cells from our active particle simulations. Figure S7 (g5-g8) & (h5-h8) show the averaged radial velocity at different time points during the aggregation. Similar to the experimental results, it can be clearly seen that during the initial stages, the z-direction separation is the dominant mode of local cellular motion, leading to an overall zero radial velocity for the both types of cells at different locations in the initial aggregation. Once the separation is achieved, both MDA-MB-231 and MCF-7 cells exhibit clear radial motion towards the center of their associated aggregations. Specifically, the radial velocities for both cell types generally increase as one moves away from the center. This is because as the cell number density increases in the central regions due to continuous in-flow of cells from the outer regions, eventually such regions become jammed and further significant cell motions are not possible. The velocity profiles are also consistent with the corresponding cell density profiles shown in Fig. S7. Initially, both types of cells are uniformly distributed and then aggregate towards the central regions until a maximal cell jamming density is achieved. We note that consistent with the experimental observations, the aggregation dynamics of the MDA-MB-231 cells including both the velocity and density profiles are synchronized with those of MCF-7 cells, indicating strongly correlated motions of these two types of cells.

**4.6 Simulated velocity correlation**


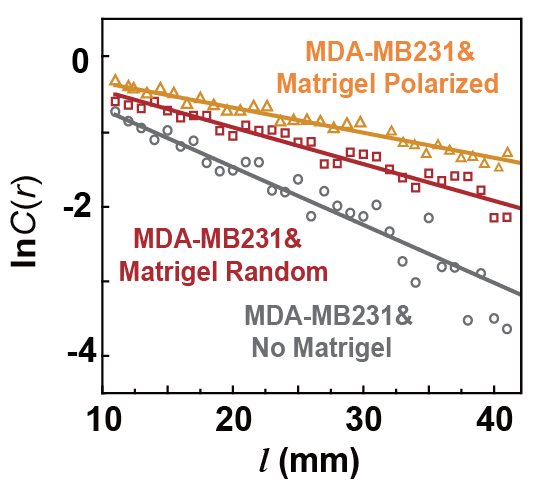


**Figure S8. Simulated velocity correlation function C(r) for MDA-MB-231 cells in different micro-environments and with different degrees of collective polarization:** the grey line with a slope of -0.078 is for the system with no Matrigel, the red line with a slope of –0.049 is for the system covered with Matrigel, in which the cells migrated almost randomly with no collective polarization, and the yellow line with a slope of -0.034 is for the system covered with Matrigel and in which cells show strong polarization motion along the z-direction. The three correlation lengths obtained by inverting the slopes are, respectively, ~12μm, ~20μm and ~29μm.

Shown in Fig.S8 is the velocity correlation function C(r) for MDA-MB-231 cells with different degrees of collective polarization and in different micro-environments, computed from the simulated cell dynamics at low cell density. Consistent with our experimental results, stronger dynamic correlations are observed for the cells in 3D Matrigel compared to those on the 2D substrate (with a correlation length a≈12µm). Furthermore, for the cases with Matrigel, the dynamic correlation between the cells in the simulated cell systems with random cell velocities (with a≈20µm) is clearly weaker than that in the systems with collective polarization along the z-direction (with a≈29µm). Note that, for the collectively polarized cells, we only computed the velocity correlations in the non-polarized directions.

**References:**

[19] Liang L, Jones C, Chen S, et al. Heterogeneous force network in 3D cellularized collagen networks[J]. Physical Biology: 2016, 13: 066001.

[20] Ma X, Schickel M E, Stevenson Mark D, et al. Fibers in the Extracellular Matrix Enable Long-Range Stress Transmission between Cells[J]. Biophysical Journal: 2013, 104: 1410-1418.

[23] Han Y L, Ronceray P, Xu G, et al. Cell contraction induces long-ranged stress stiffening in the extracellular matrix[J]. Proceedings of the National Academy of Sciences: 2018, 115: 4075.

[24] Steinwachs J, Metzner C, Skodzek K, et al. Three-dimensional force microscopy of cells in biopolymer networks[J]. Nature Methods: 2015, 13: 171.

[24] Steinwachs J, Metzner C, Skodzek K, et al. Three-dimensional force microscopy of cells in biopolymer networks[J]. Nature Methods: 2015, 13: 171.

[32] Belmonte J M, Thomas G L, Brunnet L G, et al. Self-Propelled Particle Model for Cell-Sorting Phenomena[J]. Physical Review Letters: 2008, 100: 248702.

[35] Liu L, Duclos G, Sun B, et al. Minimization of thermodynamic costs in cancer cell invasion[J]. Proceedings of the National Academy of Sciences of the United States of America, 2013, 110(5): 1686-1691.

[36] Cordelieres F P, Petit V, Kumasaka M, et al. Automated Cell Tracking and Analysis in Phase-Contrast Videos (iTrack4U): Development of Java Software Based on Combined Mean-Shift Processes[J]. PLOS ONE, 2013, 8(11): 1-10.

[37] Gorelik R and Gautreau A. Quantitative and unbiased analysis of directional persistence in cell migration[J]. Nature Protocols, 2014, 9(8): 1931-1943.
